# Protocol for a pilot effectiveness and feasibility randomized controlled trial of an advanced telerehabilitation system for people with multiple sclerosis (PLATINUMS project)

**DOI:** 10.1371/journal.pone.0355376

**Published:** 2026-08-18

**Authors:** Laurits Taul-Madsen, Lars G. Hvid, Giulia Casu, Massimiliano Pau, Eoin Synnott, Susan Coote, Yasmin Alt, Alon Kalron

**Affiliations:** 1 The Danish MS Hospitals, Ry and Haslev, Denmark; 2 Exercise Biology, Department of Public Health, Aarhus University, Denmark; 3 Department of Mechanical, Chemical, and Materials Engineering, University of Cagliari, Italy; 4 Multiple Sclerosis Society of Ireland, Dublin, Ireland; 5 Physical Activity for Health Research Centre, University of Limerick, Limerick, Ireland; 6 Department of Physical Therapy, Steyer School of Health Professions, Gray Faculty of Medical and Health Sciences, Tel Aviv University, Tel Aviv, Israel; 7 Multiple Sclerosis Center, Sheba Medical Center, Ramat Gan, Israel; University rehabilitation institute, SLOVENIA

## Abstract

**Background:**

Multiple Sclerosis (MS) causes many different symptoms, with some of the most dominant being impaired balance and walking performance. Exercise effectively improves strength, balance, and walking performance, but barriers to performing exercise, such as fatigue and logistical issues, do exist. A potential strategy to overcome these barriers is the use of telerehabilitation, which has shown promising results. Few previous studies have measured strength, balance, and walking outcomes, and none have used systems that provided real-time feedback. Therefore, the present study aims to compare telerehabilitation to a paper-based home-based exercise program to evaluate the effectiveness on strength, balance, and walking outcomes. The telehealth intervention will be delivered through an innovative platform that uses Artificial Intelligence algorithms to provide users and clinicians with tailored feedback on performance and adherence.

**Methods:**

This study is a multinational, feasibility, pilot randomised controlled trial investigating the effects of an AI-based telerehabilitation intervention in persons with multiple sclerosis (pwMS). Participants (n = 96) will be randomised to receive either 10 weeks of a home-based AI telerehabilitation intervention or a home-based exercise intervention with paper-based instructions. The primary clinical outcome is the 30-second sit-to-stand test (30STS), and secondary clinical outcomes are balance, fatigue, MS impact, quality of life, symptoms of anxiety and depression, and cost-effectiveness analyses. Feasibility, in terms of recruitment and retention, the acceptability of procedures, and compliance and adherence, will also be captured.

**Trial registration:**

ClinicalTrials.gov NCT07310914

## 1. Introduction

Multiple Sclerosis (MS) is an autoimmune, neurodegenerative disease in the central nervous system [1], affecting over 2.8 million people worldwide [2]. Many symptoms can occur during the disease course, but some of the most debilitating symptoms, according to people with MS (pwMS) themselves, are impaired lower limb strength, balance, and mobility [3,4]. Also, up to 85–90% of pwMS experience impaired balance and walking performance throughout the disease course, making these persistent symptoms [5,6]. Hence, treatments to improve lower limb strength, balance, and walking performance are highly warranted, with exercise proven to be highly effective [7].

Exercise can be conducted in various settings (supervised/unsupervised, gym-based/home-based, etc.), with supervised exercise programs at a clinic considered the most optimal and effective [8]. However, a lack of access to these programs, for instance, for those living in remote areas or where healthcare/medical facilities are available but no MS experts are present, may make it difficult to engage in optimal exercise programs [9]. In a recent survey of 741 pwMS, travel and/or moving issues were the second-largest barrier to practice exercise, behind fatigue [10].

A potential way to overcome this barrier to exercise is telerehabilitation (TR), which can be defined as “*the application of information and communications technology to deliver rehabilitation services over a distance by linking a healthcare provider to a beneficiary, caregiver, or any persons (s) responsible for delivering care to the beneficiary, for screening, assessment, intervention, consultation/coaching and/or supervision/monitoring.”* [11]

Interestingly, TR, as a concept, appears effective at improving key symptoms frequently reported by pwMS, including impaired mobility and walking performance [12,13] and impaired balance [13,14].

When focusing only on exercise-based TR, a systematic review and meta-analysis including 14 RCTs found it beneficial for fatigue, depression, and overall quality of life [15]. However, beneficial effects on balance, mobility, and walking performance were less robust and convincing.

In another systematic review investigating the effects of TR on quality of life in pwMS, TR was found to be more effective than no intervention and as effective as in-person treatment [16]. The review included 12 randomized controlled trials (RCTs), with different types of TR interventions (4 video-based interventions, 3 app-based interventions, 2 augmented reality interventions, 1 virtual reality intervention, 1 video-based intervention with focus on motor imagery, along with an app, and 1 study did not define their TR). Of the studies included, 4 had an exercise component (according to the definition by Caspersen et al. [17]) in their interventions, including pelvic floor muscle training [18,19], resistance and balance training [20], or Pilates [21].

However, despite the numerous potential benefits of TR, several limitations, such as the need for specialist equipment, complex technical solutions, technical difficulties, and lack of digital literacy, may currently prevent its widespread adoption in the MS population. Furthermore, most studies conducted have relatively small sample sizes, do not provide direct feedback to participants, and mainly report patient outcomes, with few investigating lower limb strength, balance, and walking outcomes [22].

A potential way to provide direct feedback to participants is to use a platform that applies artificial intelligence (AI). The platform (WizeCare™) used in the present study can be accessed on either a phone or a computer, with no other devices required. The platform allows direct contact between the participant and therapist using the devices’ built-in cameras. Furthermore, it will use AI software to provide direct feedback to the participant and will allow the therapist to continuously monitor adherence and compliance to the program, as well as movement quality.

Therefore, we aim to examine feasibility and preliminary effectiveness in a multinational, pilot RCT trial comparing AI TR with a paper-based, home-based exercise program. We hypothesize that the AI TR program will be superior to the paper-based exercise program in improving performance on the 30 STS test. Additionally, the RCT will include an economic evaluation to determine the cost-effectiveness of the TR system.

## 2. Materials and methods

### 2.1. Trial design

This will be a single-blind, 1:1 parallel-group, multicentre, pilot feasibility randomized controlled trial. The study protocol has been reported using the Standard Protocol Items: Recommendations for Interventional Trials (SPIRIT) statement guidelines [23], and the associated publication will be reported in accordance with the CONsolidated Standards of Reporting Trials (CONSORT) extension for randomized pilot and feasibility trials [24].

### 2.2. Trial setting

This multinational study will be conducted in Israel, Italy, Ireland, and Denmark. In Israel, it will take place at the Sheba Multiple Sclerosis Center in Tel Hashomer; in Italy, at the Regional Multiple Sclerosis Center of Sardinia (ASL Cagliari); in Ireland, at the MS Society of Ireland, Dublin; and in Denmark, at the Danish MS Hospitals in Ry and Haslev.

### 2.3. Eligibility criteria

*Inclusion criteria*

Participants must meet all the following criteria to be eligible for the study:

[a] Age 18 years or older.
[b] Definite diagnosis of multiple sclerosis (MS) according to the McDonald criteria [25] (including both relapsing–remitting and progressive forms).
[d] Expanded Disability Status Scale (EDSS) score between 2.5 and 6.5, or Patient Determined Disability Score (PDDS) between 2 and 6, corresponding to deficits in lower-limb functional performance
[e] Preserved ambulatory function, defined as the ability to walk at least 20 meters independently, with or without an assistive device.
[f] Willingness and ability to travel to the local study site for evaluation sessions.
[g] Access to an internet-enabled platform suitable for study participation.

*Exclusion criteria*

[a] Presence of comorbidities that may hinder safe participation in the study.
[b] Documented MS relapse and/or corticosteroid treatment within the past three months.
[c] Severe cognitive impairment that prevents following simple instructions or providing informed consent.

### 2.4 Recruitment

Participants will be recruited through specialized inpatient and outpatient MS clinics and via social media platforms commonly used by pwMS. Prior to enrolment, potential participants will undergo an in-person screening to determine eligibility based on the study’s inclusion and exclusion criteria. Eligible individuals will receive oral information about the study and will be allowed to ask questions. In addition, they will be provided with two information leaflets: one describing the study’s rationale, design, and objectives, and another outlining their rights as participants in a health science research project.

### 2.5. Randomization

Participants who meet all eligibility criteria and complete the baseline evaluation will be randomly assigned at each site in a 1:1 ratio to either the TR intervention group or the paper-based exercise group. A computer-generated randomization sequence stratified by study site will be used. Randomization procedures at all sites will be coordinated according to a common study protocol developed by the project team. Allocation concealment will be ensured using sequentially numbered, opaque, sealed envelopes prepared and maintained locally at each participating site. Envelopes will be opened only after eligibility has been confirmed and baseline assessments have been completed.

### 2.6. Intervention and comparator

#### Overview.

Participants will be randomised to one of two home-based exercise programs while continuing to receive their usual care: (1) an AI-supported TR intervention or (2) a paper-based exercise program serving as the comparator. The intervention period for both groups will be 10 weeks, and in line with current recommendations [26], participants will attend two sessions per week, each lasting 30–45 minutes, for a total of 20 sessions per participant.

Prior to the first at-home session, an in-person physiotherapy assessment will focus on the personalized exercise prescription and detailed instruction on the assigned delivery method (AI TR or paper-based).

Adherence and compliance with the exercise prescription will be monitored throughout the intervention period using an exercise logbook that captures details based on the FIIT-VP principles (frequency, intensity, time, type, volume, and progression) [27].

As usual, care varies across participating countries; all participants will be asked to complete a diary documenting any exercise or physical activity performed outside the study.

Every week, each participant will receive a text message to address technical issues, monitor for adverse events, remind them to fill out their log, and motivate them. In weeks two and six of the project, each participant will receive a phone call to adjust the exercise program accordingly. To promote retention, participants will receive regular contact throughout the study, including weekly text messages and scheduled telephone calls. Flexible scheduling of assessments, individualized exercise progression, and technical support for the telerehabilitation platform will be provided as needed.

#### Intervention: Telerehabilitation (TR) Program.

The telerehabilitation arm will utilize the AI-driven Wizecare^TM^ digital platform (https://www.web.wizecare.com/platform), a commercially available system designed for remote physical therapy and home-based exercise training. WizeCare^TM^ provides access to over 800 exercises (103 Move AI exercises) that target the strength, balance, coordination, and flexibility of the lower limbs, upper limbs, and core. Exercises will be prescribed by physiotherapists according to evidence-based guidelines for individuals with MS [28] and the physiotherapist’s clinical reasoning, and will include approximately 8–10 exercises tailored to the individual’s functional status and needs.

To ensure consistency across sites, exercise prescription will follow a common intervention framework developed by the consortium. Exercise programs will include a combination of lower-limb strengthening, balance, and mobility exercises, with individual tailoring based on participants’ functional status. Progression of exercises will be guided by participant performance, perceived exertion, and safety considerations. Prior to study initiation, all physiotherapists will receive training on the study procedures and intervention framework. Intervention fidelity will be supported through regular consortium meetings and periodic review of exercise prescriptions and participant logs across sites.

A key technological component of the system is MoveAI (patent no. US 11,636,777 B2), which enables motion detection, tracking, and analysis through the built-in camera of a smartphone, tablet, or PC. This allows real-time feedback and guidance without the need for dedicated wearable sensors. The platform complies with international security and privacy standards, including ISO/IEC 27001:2013, ISO 27799:2016, and AMAR 29460001.

Using computer vision technology, MoveAI tracks participant movements during exercise and compares performance against predefined movement parameters. The system provides real-time feedback regarding exercise execution, movement quality, range of motion, and repetition completion, thereby supporting independent exercise performance. In addition, exercise completion and engagement metrics are automatically recorded, allowing clinicians to remotely monitor adherence and progression. AI-enabled telerehabilitation systems have emerged as a promising approach to enhance accessibility, personalization, and monitoring of rehabilitation interventions delivered in the home environment.

Participants will perform asynchronous home-based exercise sessions without the physiotherapist’s input. Ongoing adherence and compliance will be captured automatically through the WizeCare platform and through the written exercise logbook, where participants note the amount of exercise performed and rate their perceived exertion.

#### Comparator: Paper-Based Exercise Program.

Participants randomized to the comparator group will receive a paper-based, individualized home exercise program immediately after their baseline assessment. As in the TR arm, exercises will be prescribed according to evidence-based guidelines for individuals with MS and the physiotherapist’s clinical reasoning and will include approximately 8–10 exercises tailored to the individual’s functional status and needs. Participants will conduct all exercise sessions independently at home, following the written instructions. Adherence will be monitored through the written exercise logbook, where participants note the amount of exercise performed and rate their perceived exertion.

### 2.7. Outcomes

The ***primary clinical outcome*** of the trial is lower-limb functional performance, assessed using the 30-Second Sit-to-Stand Test (30STS). This test was selected as it represents a weight-bearing activity relying on balance, coordination, and muscle strength/power (predominantly lower extremity). Moreover, the majority of exercises in the trial target lower-limb muscle groups similarly. During the 30STS, participants rise from a standard chair without armrests as many times as possible within 30 seconds, with the total number of repetitions recorded as the score. The 30STS has demonstrated strong validity and reliability in pwMS [29]. The primary outcome will be assessed at baseline (T0) and at the end of the 10-week intervention period (T1).

To characterize the study sample, a set of descriptive and clinical measures will be collected at T0, including sex, age, height, body mass, time since MS diagnosis, MS subtype (i.e., relapsing–remitting, secondary progressive, or primary progressive), medication use, comorbidities, disability status (EDSS or PDDS), and use of walking aids (cane, crutches, or rollator). Digital literacy will also be assessed at baseline using the Digital Literacy Scale [30].

***Secondary clinical outcomes*** will be assessed at T0 and T1. Mobility and balance will be evaluated using the Short Physical Performance Battery (SPPB), consisting of standing balance tests, walking speed, and repeated chair rises [31,32], the MiniBESTest [33–35], and the Functional Reach Test (FRT) [36,37]. Walking performance will be measured using the Timed 25-Foot Walk Test (T25FWT) [38,39] and the Six-Minute Walk Test (6MWT) [40]. Cognitive function will be assessed with the Symbol Digit Modalities Test (SDMT) [41].

A range of patient-reported outcomes will be collected at both T0 and T1. These include the MSIS-29 for the physical and psychological impact of MS [42], the Modified Fatigue Impact Scale (MFIS) for fatigue [43], the Multiple Sclerosis Walking Scale–12 (MSWS-12) for perceived walking limitations [44], the State-Trait Anxiety Inventory (STAI) for anxiety [45], the Quick Inventory of Depressive Symptomatology (QIDS) for depressive symptoms [46], and the EQ-5D-5L for health-related quality of life [47]. All participants will complete a semi-structured interview exploring usability, satisfaction, and experiences with the trial process, providing qualitative insights that complement the quantitative outcomes.

An ***economic evaluation*** will be performed from both health-system and societal perspectives. The economic evaluation methodology, data collection procedures, and resource-use measures were developed with input and guidance from a health economics expert within the PLATINUMS consortium to ensure the appropriateness of the proposed approach. Participant-level resource use, including healthcare and social care contacts, informal caregiving, productivity loss, medication use, assistive devices, and out-of-pocket expenses, will be assessed using an adapted Client Service Receipt Inventory (CSRI) [48]. Provider and system-level costs (e.g., platform setup, operational resources, staff training, therapist time, clinical space, administrative support, printed materials) will be captured using standardized implementation cost forms. Quality-adjusted life years (QALYs) will be derived from EQ-5D utilities using the area-under-the-curve method, and costs and QALYs will be compared between study arms to compute the Incremental Cost-Effectiveness Ratio (ICER) [49].

Feasibility of the trial. To assess the feasibility, we will evaluate 1) recruitment and retention rates, 2) evaluation of data collection procedures, 3) evaluation of acceptability of the interventions and study procedures, 4) evaluation of the ability of the consortium to manage the study, 5) safety of the interventions, and 6) preliminary measures of effectiveness of participant responses to the intervention.

When evaluating recruitment rates, we will determine whether 8 months is an acceptable time frame for recruiting 96 participants across 4 sites. To evaluate the data collection and intervention procedures, our semi-structured interviews with participants in each country will help us determine them. For the intervention’s acceptability, we consider an 85% retention rate acceptable and will explore this further in the semi-structured interviews. Furthermore, we will use the Telehealth Usability Questionnaire (TUQ) [50] to assess the usability of the TR system. Trial management will be overseen by an external advisory board. Adverse events (AEs) will be closely monitored throughout the study period, and rates will be compared with those in other exercise intervention studies [51].

**Fig 1:**
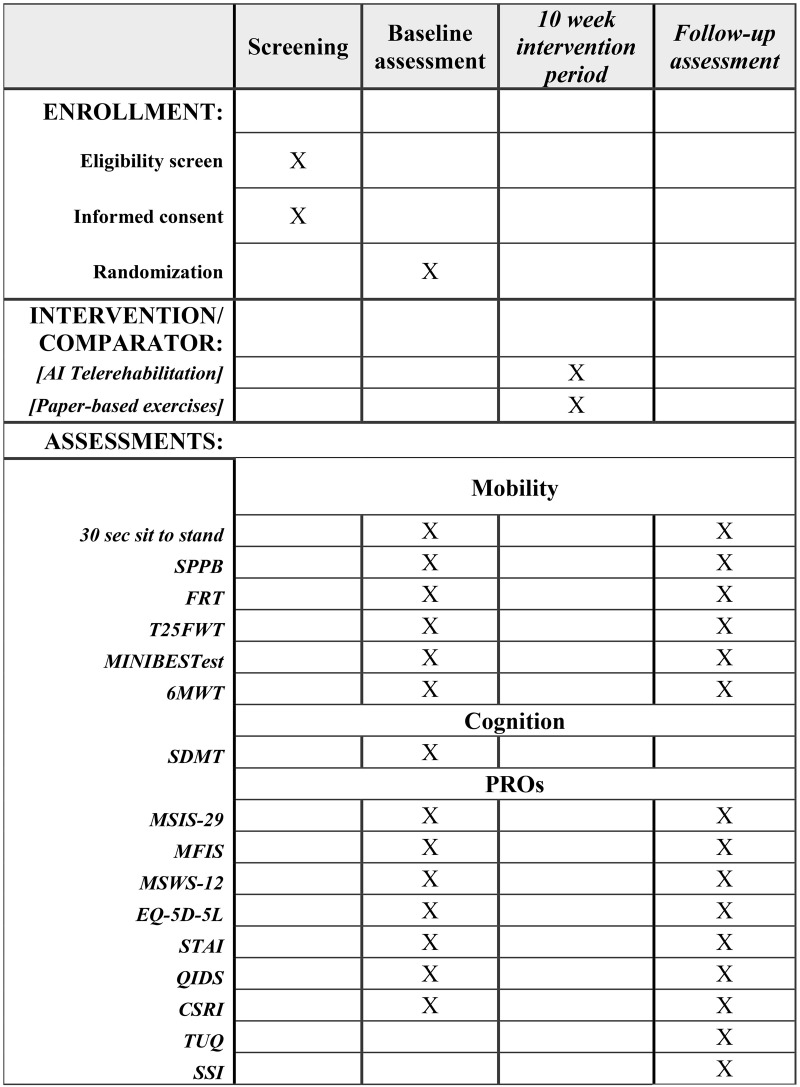
Participant timeline- schedule of enrollment, interventions, and assessments. Abbreviations: SPPB: Short Physical Performance Battery, FRT: Functional Reach Test, T25FWT: Timed 25 Feet Foot Walk Test, MINIBEStest: Mini Balance Evaluation Systems Test, 6MWT: Six-minute Walk Test, SDMT: Symbol Digit Modalities Test, MSIS-29: 29-Item Multiple Sclerosis Impact Scale, MFIS: Modified Fatigue Impact Scale, MSWS-12: 12-Item Multiple Sclerosis Walking Scale, EQ-5D-5L: EuroQol 5 Dimensions 5 Levels, STAI: State-Trait Anxiety Inventory, QIDS: Quick Inventory of Depressive Symptomatology, TUQ: Telehealth Usability Questionnaire, SSI: Semi-structured interview.

Finally, the study will assess multiple outcomes to evaluate the intervention’s preliminary effect, and adherence of ≥ 80% and compliance with the intervention of ≥ 75% are considered acceptable to proceed to a full trial (Fig 1) [52].

### 2.8. Harms and Adverse Events

Although no significant risks are anticipated in this study, the TR group may have a higher risk than the group doing exercises based on paper-based instructions. Also, as approximately 50% of pwMS experience falls, it cannot be omitted that some participants may fall during the intervention period [53]. The exercise programs included in both intervention arms consist of movements commonly used in physiotherapy for pwMS and are not expected to cause harm. For participants new to exercise and/or being deconditioned due to long-term physical inactivity, transient muscle soreness may occur, particularly the day after training. This response is considered normal and is not regarded as an adverse event.

To minimize the risk of falls or injury during home-based practice, the physiotherapist will provide individualized instructions on how to arrange a safe exercise environment, including guidance on supports, suitable flooring, adequate space, and removal of potential hazards. Participants will also be encouraged to take rest breaks as needed, especially in cases of fatigue or overheating, and to stop exercising if they feel unwell. All adverse events, regardless of severity or suspected relation to the intervention, will be systematically recorded and monitored throughout the study.

### 2.9. Sample size

As this study is a pilot feasibility RCT, one aim is to assess feasibility, refine study procedures, and obtain preliminary estimates of variability to inform sample size for a future definitive RCT. Accordingly, the sample size is based on methodological guidance for pilot studies rather than on a formal power calculation. Following the stepped rule-of-thumb proposed by Whitehead et al.[54], approximately 12 participants per arm (i.e., 24 participants in total) are recommended for early-stage pilot trials with continuous outcomes and small to medium expected effect sizes. In line with this guidance, 24 participants per country (a total of 96) will be recruited for the study. If needed, data can be analyzed separately by country. This sample size is expected to provide sufficient information on feasibility metrics, acceptability of study procedures, and change in the primary outcome.

### 2.10. Assessors and blinding

Outcome assessments at all study sites will be conducted by physiotherapists with at least 2 years of clinical experience with pwMS and prior familiarity with the functional tests included in the protocol. To ensure consistency and standardization across countries, a test manual has been developed detailing all assessment procedures. All assessors will receive instruction and training to perform each test strictly in accordance with this manual. Outcome assessments at both T0 and T1 will be conducted by assessors blinded to group allocation whenever possible. Participants will be instructed not to disclose their treatment allocation during assessments. Standardized assessment procedures will be used across sites.

Any instances of unblinding will be documented.

### 2.11. Data collection, management, and analysis

All study data will be entered directly into a consortium Microsoft SharePoint site, which will serve as the secure, central data repository for the project. SharePoint will be hosted by the University of Limerick, with controlled access provided to all sites. A comprehensive Data Management Plan and Joint Controller Agreement have been developed and signed by all partners prior to study commencement, outlining procedures for data entry, storage, access rights, confidentiality, data protection, and quality assurance. Regular data checks will be performed to ensure completeness and accuracy. Data quality monitoring will be coordinated centrally by S.C., who leads the project’s data management work package. Regular consortium meetings and Trial Management Committee/Advisory Board meetings will be held to review data quality and protocol adherence across all participating centers. All procedures will be conducted in accordance with the project Data Management Plan and Joint Controller Agreement. Data for each country will be held locally using relevant procedures and pseudonymized data will be entered into the consortium SharePoint platform held by UL, which serves as the secure central repository for the study. Monthly reviews will be conducted to identify missing data, inconsistencies, out-of-range values, and protocol deviations. Any queries will be communicated to the relevant site for clarification or correction against source. The study will comply with the FAIR data guiding principles [55].

#### Statistical Analysis Plan.

Feasibility outcomes, including recruitment rate, retention rate, adherence, compliance, acceptability, and adverse events, will be summarized using descriptive statistics. Continuous variables will be reported as means and standard deviations or medians and interquartile ranges, as appropriate, while categorical variables will be reported as frequencies and percentages. These outcomes will be used to assess the feasibility of conducting a future definitive trial.

Baseline demographic and clinical characteristics will be summarized using appropriate descriptive statistics. Variables with a normal distribution will be reported as mean and standard deviation, while non-normally distributed variables will be summarized as median and interquartile range. To examine changes in outcomes from baseline to the end of the intervention period, a multivariate repeated-measures mixed-effects regression model will be applied. Participant ID will be included as a random effect, and group (intervention vs. comparator) and time (T0 vs. T1) will be included as fixed effects. The model’s assumptions, including normality of residuals and homogeneity of variance, will be assessed using graphical and statistical methods. If these assumptions are not met, appropriate data transformations, non-parametric methods, or other alternative statistical approaches will be considered.

The primary analysis will adhere to the intention-to-treat principle, ensuring that all randomized participants are analyzed in the groups to which they were originally assigned, irrespective of adherence or protocol deviations. A secondary per-protocol analysis will be conducted, including participants who complete the post-intervention assessment and meet the predefined adherence (≥80%) and compliance (≥75%) criteria. Sensitivity analyses will be performed to assess the impact of missing outcome data and to compare findings obtained from the intention-to-treat and per-protocol populations. Robustness will be evaluated by examining whether the overall direction and interpretation of the results remain consistent across these analyses.

### 2.12. Stakeholder and Patient Involvement

An external advisory board will provide independent scientific oversight and trial management. Furthermore, each site has established a patient panel of 3–4 pwMS, who have guided the development of this protocol and will provide feedback on recruitment material. Following study completion, the patient panels will be actively involved in discussions about the overall feasibility and effectiveness, and whether to proceed to a full trial. Additionally, they will advise on and be part of the dissemination to ensure that results are communicated clearly and meaningfully.

### 2.13. Ethical consideration, declarations, and dissemination

This study protocol adheres to the Standard Protocol Items: Recommendations for Interventional Trials (SPIRIT) guidelines. All participating sites have obtained approval from their respective ethics committee (Israel: Sheba Medical Center IRB Journal no. SMC-2730-25, Ireland: Education and Health Sciences Research Ethics Committee Journal no. 2025_01_17_EHS, Italy: Comitato Etico Sardegna Prot Journal no. Prot. 21448, Denmark: Journal no. 1-10-72-196-25. The study is registered at ClinicalTrials.gov (NCT07310914) and adheres to the principles outlined in the Declaration of Helsinki and its later amendments. All participants will provide written informed consent using forms approved by the local ethics committees. Results from the study will be disseminated in international peer-reviewed journals, regardless of whether the findings are positive, negative, or neutral.

### 2.14. Current trial status

Recruitment has not yet begun. It is expected that recruitment will be completed by the end of 2026 and that data collection will be completed early in 2027. Results are expected to be published in 2027.

## 3. Discussion

This protocol describes a pilot feasibility RCT designed to evaluate an advanced TR intervention incorporating MoveAI for pwMS.

### Strengths and Limitations

A major strength of the study is the novelty of the intervention (details on the MoveAI features have been outlined above). If found feasible, the approach may help overcome access barriers for pwMS who have difficulty attending in-person rehabilitation and extend the effects of intensive rehabilitation/exercise interventions administered through national health systems, but that are limited in time due to budget constraints. Another strength is the integration of both patient-reported outcomes and objective system-generated mobility data, providing a multidimensional understanding of how the TR system and training program may function in real-world settings. The study also includes a relatively large sample (n = 96) for a pilot RCT across multiple international sites, enhancing the robustness of the feasibility and effectiveness metrics and later implementation into practice.

Importantly, several limitations should also be acknowledged. As a TR intervention, technical challenges, including device compatibility, network connection stability, and varying levels of digital literacy, can affect the delivery of the intervention and participant engagement. These challenges will be monitored throughout the trial and reported as part of the feasibility outcomes. Physical activity outside the intervention will be assessed using self-reported diaries, which may introduce recall bias; objective activity trackers would provide complementary real-world data, but their use is beyond the scope of this study. Finally, as a feasibility trial, the study is not powered to detect efficacy, and any observed clinical trends will be interpreted cautiously.

### Operational Considerations, Amendments, and Dissemination

Because this study involves sites from different countries and operational systems, differences in site logistics, staffing, and local clinical practices may influence recruitment rates and adherence, and hence each country has sufficient numbers for its own pilot study analysis. Standardized training and regular cross-site communication will be used to minimize variability. Any protocol amendments will require approval from the consortium, the advisory board of three MS rehabilitation experts, and all relevant institutional ethics committees. The study may be paused or terminated if major unforeseen safety or operational concerns arise.

Findings from this feasibility trial will inform the design of a future fully powered RCT. Regardless of outcome (positive, negative, or neutral), results will be disseminated through peer-reviewed publications, conference presentations, and patient-focused communication materials co-created with patient panels at each site.

### Perspectives

Although feasibility outcomes must be interpreted within the scope of an early-stage evaluation, the successful implementation of MoveAI could represent a meaningful advance in MS TR. Beyond supporting exercise participation and motivating adherence and compliance, continuous tracking of movement patterns may eventually help identify subtle functional decline and inform more personalized, timely rehabilitation strategies.

## Supporting information

S1 File Protokol_version2_cleanversion. (PDF)

S2 File SPIRIT 2025 participant timeline. (DOCX)

S3 File SPIRIT checklist. (DOCX)
