# Supplementary material for: Protocol for a pilot effectiveness and feasibility randomized controlled trial of an advanced telerehabilitation system for people with multiple sclerosis (PLATINUMS project)
Source: PLoS One. 2026 Aug 18;21(8):e0355376. doi: 10.1371/journal.pone.0355376 (PMC13485066; doi:10.1371/journal.pone.0355376)
Supplement: Supplementary file 1 [file pone.0355376.s001.pdf]

Protokol, version 2 15/01-26

Title
Pilot feasibility RCT of an advanced telerehabilitation system in people with multiple sclerosis

Danish title
Et randomiseret kontrolleret pilotstudie, der undersøger gennemførligheden af et avanceret telerehabiliteringssystem på personer med multipel sklerose.

Purpose
The overall purpose of the present study is to investigate the feasibility, acceptability and preliminary effects of an AI-powered telerehabilitation system on mobility outcomes compared to paper-based home exercise instructions. Furthermore, the study aims to conduct an economic evaluation of the telerehabilitation system and intervention programs based on the pilot feasibility RCT data.

Introduction
Multiple sclerosis (MS) is a chronic, progressive neurodegenerative disease of the central nervous system, affecting over 2.8 million people worldwide(1). One of the most common problems reported by people with MS (pwMS) is impaired mobility, specifically the inability to walk safely and independently(2). Approximately 85-90% of pwMS report mobility deficits during the disease(3), which can commence very early after onset(4). Most pwMS will eventually require assistance i.e., a cane or walker, due to disease progression(5). In other cases, walking impairment will become severe enough for one to require a wheelchair or scooter. Decreased mobility leads to less physical activity(6); physical inactivity thus, leads to and perpetuates deconditioning, which in turn will lead to further impairment of mobility(7). At some critical point, mobility will be limited to such an extent that there is irreversible disability resulting in secondary health conditions that will be difficult, if not impossible, to treat(8). This progression of disability can result in a deterioration of mood, which will further contribute to decreased social, recreational, and vocational participation in society and ultimately produce a poor quality of life(9). The decrease in participation and work productivity taken in combination with an increase in secondary health conditions and medical needs significantly impacts global economy worldwide(10). MS is the leading cause of disability in young and middle-aged adults. The life expectancy for pwMS approaches that of the non-disabled population(11), thus, a reduction in physical activity is of significant social and economic importance. Mobility must be maintained and measures to combat or reverse this downward spiral and progression of disability

must be developed and implemented to reduce the burden of this disease not only on pwMS and their families, but society in general.

Exercise can improve mobility and perhaps decrease the rate and extent of disability (i.e. impaired function) in pwMS(12, 13). Hospital/clinical facility-based exercise programs particularly with other pwMS or under the supervision of MS experts, may help pwMS to participate in exercise or exercise at a higher, more intense level(14). However, lack of access to these programs, especially, if one lives in a remote area where there are few options, or where there are health care/medical facilities but no MS experts, may make it difficult to engage in a facility-based exercise training program(15). The COVID-19 pandemic highlighted the need to find appropriate solutions to enhance exercise and physical activity in the MS population. In a series of studies involving members of the present consortium, we reported that physical activity and exercise activities, especially at moderate and high intensities, decreased during the pandemic in pwMS(16, 17). Moreover, the research consortium has strongly concluded that to ensure the continuity of exercise training for pwMS during future pandemics, healthcare stakeholders are highly recommended to provide training via advanced remote technologies concurrently with professional guidance and support(18).

Telerehabilitation (telerehab) has great potential for providing accessible and cost-effective options for exercise in the home environment for pwMS, particularly if there is ongoing communication with knowledgeable exercise personnel who ensure safe exercising. Only a few studies have tested telerehabilitation as a way to enhance exercise and physical activity in pwMS, and found positive results(19, 20). Nevertheless, despite the advantages of this treatment strategy, there are still a number of limitations that prevent the use of the technology on a large scale. The existing solutions are either too simplified, do not provide feedback when the patient does not perform the exercise correctly, or requires special hardware (e.g., movement sensors) that suffer from malfunctions and in several cases poor technical support.

In the present project, we intend to implement a cutting-edge artificial intelligence (AI) technology for a home-based exercise therapy program for pwMS. One of the advantages of this system includes its technical simplicity. The system requires nothing but a mobile phone, tablet, or a personal computer and no need for any additional 3rd party hardware of any kind. This feature is extremely important for pwMS who commonly suffer from cognitive deficits. Moreover, the technology uses an AI algorithm (based on range of motion and speed) enabling real time feedback as well as positive encouragement during practice. The system also includes over 800 exercise drills that can be customized by the therapist according to his/her patients' goals and needs. The

revolutionary telerehabilitation technology (https://www.wix.wizecare.com/) has been implemented in numerous healthcare services with thousands of users (mainly in the field of orthopaedic rehabilitation), and has exhibited encouraging results in conjunction with positive feedback from patients, physical therapists, and stakeholders. Nonetheless, it has never been implemented in the MS population.

Therefore, our overall goal is to investigate the feasibility of an innovative telerehabilitation exercise training system for pwMS, through assessment of its efficacy on mobility. For this primary aim, we propose a pragmatic multicentre trial in established MS centres across the EU community (Denmark, Italy, Israel and Ireland) investigating the feasibility of exercise delivery via telerehabilitation. A key aspect of this project is the inclusion of severely disabled pwMS (wheelchair users). To date, this sector of pwMS has been largely ignored in MS exercise projects. We believe that for these individuals, a telerehab exercise solution must be promoted. Since many live-in relative isolation and/or have difficulties reaching a hospital/clinical based training facility, remote home-based solutions may possibly be their only solution.

Furthermore, we aim to validate a set of remote mobility clinical tests delivered via the telerehabilitation system with the purpose to evaluate balance and functional mobility in pwMS.

Methods

Study design

The present study will be a pilot feasibility randomised controlled trial investigating exercise delivery via telerehabilitation. Participants will either receive the intervention (home based telerehabilitation) or home based exercise delivered via paper-based instructions. The study is a multicentre study conducted in four countries (Denmark, Italy, Israel and Ireland), and will, in Denmark, be conducted at the Danish MS Hospitals.

Participants

Participants in the present study will be included according to the following criteria:

- Adults 18 years or older with a confirmed diagnosis of MS based on the McDonald criteria(21).
- An Expanded Disability Status Scale (EDSS) of 2.5 to 6.5 or an equivalent Patient Determined Disability Score (PDDS, indicating a preservation of at least some ambulatory function, that is, the ability to walk 20 meters or more, independently, with or without the use of an assistive device.
- Must be willing to travel to the local study site for the evaluation sessions.
- Internet platform availability.

Participants will be excluded according to the following criteria:

- Another neurological, orthopaedic, or cardiovascular condition interfering with walking.
- Individuals who have had a documented relapse and/or corticoid treatment during the past three months.
- Individuals with severe cognitive deficits who cannot follow simple instructions and/or consent.

Sample size

For the present feasibility study, a total of 96 participants will be included (24 at each site). As this is a feasibility study, the sample size is based on previous proposals stating that the sample size should be based on "practical considerations including participant flow, budgetary constraints, and the number of participants needed to reasonably evaluate feasibility goals."(22)

Interventions

Telerehab

Participants who will receive the telerehabilitation intervention, will have an exercise program prescribed through the telerehabilitation system. The telerehabilitation system is based on an AI telerehabilitation platform (Wizecare™). This is a digital-health medium-sized company that has developed an innovative solution for remote physical therapy and exercise training at home. The system includes over 800 exercises targeting the lower and upper body and core muscle groups,

hereby enabling the therapist to make personalised plans. Moreover, the system includes a technology entitled MoveAI™ (patent no. US 11,636,777 B2) which identifies, tracks and analyses the user's movement without needing sensors, wearables, or depth cameras.

This will provide the patient with immediate feedback and guidance. The system is safe and has been confirmed by several security certificates of the highest standard (ISO/IEC 27001:2013; ISO27799:2016; AMAR (Israeli standard):29460001).

Following an initial guidance session, participants will perform 20 home-based training sessions, each lasting 30-45 minutes, over a ten-week period resulting in two sessions per week.

Paper-based

Participants receiving the paper-based intervention will, following the baseline intervention, be handed a paper-based exercise program meant to be performed at home. The program will be personalised and made by an experienced physiotherapist, and will match the Telerehab program in frequency (20 sessions over a 10 week period).

Outcomes

Demography

To describe the participants, information on sex, age, height, weight, time since MS diagnosis, type of MS (relapsing-remitting, secondary or primary progressive), use of disease-modifying drugs, EDSS or PDSS and use of a walking aid will be collected.

Patient-reported outcomes

To assess the participant's perception of the telerehabilitation, the Telehealth Usability Questionnaire (TUQ) will be used. This is a solid, robust and versatile tool to measure the quality of a telehealth's system's interaction and services(23). Also, a semi-structured interview with the participants and physiotherapists involved in the project will be carried out, to learn on the usability and satisfaction with the telerehabilitation system. In regard to the answers of patient reported outcomes, these are only used as outcomes in the study and not in a clinical context. In connection with hospital admission, patients complete a series of questionnaires, which are specifically used to screen for depressive symptoms and similar conditions, and are applied in a clinical context.

In order to measure the patient's own perception of their walking ability, the 12-time Multiple Sclerosis Walking Scale (MSWS-12) will be used(24). To measure quality of life, the EQ5D-5L(25) and the Multiple Sclerosis Impact Scale (MSIS29 v.2)(26) will be used. To measured the level of perceived fatigue, the Modified Fatigue Impact Scale (MFIS)(27) will be used. To measure anxiety, the Trait State Anxiety inventory (STAI)(28) will be used, and to measure depression the Quick Inventory of Depression Symptomology (QIDS)(29) will be used.

Economic evaluation

To evaluate the potential economical benefit of the telerehabilitation system, the Client Service Receipt Inventory (CSRI)(30) will be used.

Balance and mobility test

To assess balance and mobility, participants will perform the 30sec STS(31), the miniBESTest(32) and functional reach test (FRT)(33). The Short Physical Performance Battery (SPPB), consisting of the side-by-side stand, semi-tandem stand, tandem stand, a gait speed test on 3 metres and a repeated chair stand test, will be used to assess physical performance(34). To measure gait ability, the timed 25 feet foot walk test (T25FWT) and the Six-minute walk test (6MWT) will be used(35).

Cognitive function

To measure cognitive function, processing speed, the Symbol Digit Modalities Test will be used (SDMT)(36).

Safety

To assess the safety of the innovate telerehabilitation, the occurrence of adverse event will be reported.

Discussion and perspectives

The present study is a pilot feasibility RCT, with the purpose to test the feasibility and preliminary effects of telerehabilitation in pwMS.

If successful, the multicenter RCT has a great potential to improve the health of pwMS**.** If improvements in mobility outcome measures in the telerehabilitation exercise group will resemble those of participants in a facility-based physical therapy group, high quality exercise training to improve mobility can be provided remotely. Consequently, more pwMS will be able to receive guided exercise training, as travel to an exercise facility will not be needed.

We note that while telerehabilitation may not have been explicitly mentioned in the Rehabilitation 2030 initiative (under the World Health Organization; WHO), the WHO initiative does emphasize the importance of improving access to rehabilitation services, especially for those will live in remote or isolated places. The project has the potential to improve access to rehabilitation for many pwMS and other neurological conditions.

To better understand our enthusiasm of increasing exercise training in pwMS, the importance of exercise for pwMS must be reiterated. Lack of exercise leads to and perpetuates deconditioning, which in turn will lead to further impairment of mobility. At some critical point, mobility will be limited to such an extent that there is irreversible disability resulting in secondary health conditions that will be difficult, if not impossible, to treat. This progression of disability can result in apathy and depression, further contributing to decreased social, recreational, and vocational participation and ultimately a poor quality of life. The decrease in participation and work productivity taken in combination with an increase in secondary health conditions and medical needs have a significant economic impact_in Europe. Since MS is the leading cause of disability in young and middle-aged adults, and the life expectancy for pwMS approaches that of the non-disabled population, the reduction in mobility is of significant social and economic importance. Of importance, the burden of MS is not only on pwMS and their families, but society in general. Therefore, the expected impact of the project is through reversing the spiral deterioration resulting from lack of exercise.

The project is expected not only to increase exercise activity in pwMS, but also change the traditional way of monitoring home based self-exercising. Traditionally, once the therapist wishes his/her patient to self-practice exercise at home, he/she usually hands over an exercise sheet, detailing the requested exercises. In several cases the therapist provides to the patient access to demonstrations presented via video clips. The traditional way does not allow the therapist to know for real whether the patient is actually following his/her recommendations. Furthermore, there is no way for the therapist to know if the exercises are performed correctly and safely. The project solves this problem through an

innovative approach using a modern and unique technological solution. The telerehabilitation system to be studied monitors exercise performance of the complete exercise program as well as for the individual exercise. In case where the patient does not follow the exercise program, the therapist receives an alert requesting him/her to contact the therapist in order to find out the reason for not training. An additional innovative feature of the telerehabilitation system is its ability to track in real time the exercise performance by the patient, with emphasis on quality of movement, a feature unique to the Wizecare system. In case where the patient performs the exercise wrongly, he/she receives an immediate feedback and instructions on how to perform the exercise correctly.

We are confident that the collective features of the innovative telerehabilitation system can improve dramatically the traditional way home-based exercise is delivered and is expected to play an increasingly prominent role in delivering accessible, convenient, and effective rehabilitation care in the near future.

Risk, side effects and disadvantages

Overall, this project is risk-free for participants fitting the inclusion criteria. Minimal risk for side effects is present during and following the intervention. There is a low possibility that the telerehabilitation system is reported not feasible, as it is currently in use with thousands of satisfied users. Nevertheless, this issue can be solved by modifying various features of the system in line with comments raised by the participants.

In general, research participation poses minimal risk to subjects. Participants will receive the exercise intervention exercise via Telerehab or a paper-based program. There is a minimal risk that exercise may cause muscle soreness or a brief increase in fatigue. The risk of relapse is minimal. If a participant experiences ongoing MS symptoms, they may be dropped from the study. Completion of questionnaires and clinical outcome measures will pose little if any risk; however, some participants may become fatigued during the completion of these measurements. In any case, adverse events will be documented and reported. Some of the questions we will ask as part of this study may make some participants feel uncomfortable. Selective refusal of any questionnaires that participants find sensitive will be permitted to avoid psychological stress.

The potential risks of participating in a project involving a web-based intervention tool includes identity theft, electronic fraud/security breaches, electronic monitoring, stalking or bullying, hacking, and phishing. The telerehabilitation system will collect certain types of identifying information including names and email addresses from participants. Direct identifiers will not be shared with

others; however, the principal investigator will be sharing the demographic (age, height, weight etc.) and health-related information (disease status and medication) provided by participants with the other partners from Italy, Ireland and Israel, and it is possible that in some instances, this information may be sufficient to uniquely identify an individual. It is also possible that through a breach in security, a participant's identifying information could become known in conjunction with their participation in the study.

Information from patient journals

To identify participants, the visitation department of the MS hospitals screen (based on journal information for the last year) for participants with an age above 18 and an EDSS of 2.5-6.5. All participants screened for this project are referred to the MS hospitals by their neurologist or general practitioner. This information is passed on to the trial manager, along with their name and phone number, allowing him to make contact. It is important to note, that only age, EDSS, their name and phone number are granted to the researchers before participants have signed informed consent. It is estimated to concern 50 persons, in according to desired sample size. Potential participants will be contacted in person by their contact-person at the hospital, who will briefly introduce them to the project. If interested, the trial manager will have a more in depth conversation with the potential participant regarding the project.

After signed consent, information on MS phenotype, time since diagnosis, and use of MS-specific medication will be collected from the participants' patient journals. This information will be used to describe the population properly and will be gathered throughout the study period.

The consent will grant the trial manager and any regulatory authorities direct access to retrieve information from the patient's medical records, including electronic health records. This access is necessary to obtain information about the participant's health status, which is required for the conduct of the research project as well as for control purposes, including self-monitoring, quality control, and monitoring, which the authorities are obliged to perform.

Handling of personal information

The trial will be registered at ClinicalTrials.gov. All data collected is handled confidentially and stored in accordance with the current regulations, including GDPR and the Danish Data Protection Act (LBK no. 289 á 08/03/2024)

Persons from the authorities who supervise health science research projects must have access to all data if they carry out an inspection of the experiment. The subjects are informed of this and, by their

consent to participation, also give permission for these subjects to have access to the subjects' data. Officials are subject to a duty of confidentiality. Should the subject choose to withdraw their consent during the experiment, data collection and intervention will stop immediately, but participants will be made aware that the researchers can include data collected up until the withdraw date in the overall data analysis. The subject's data and samples will be processed pseudonymised, i.e., name or other personally identifiable information will not appear in any of the material, which will instead be marked with a participant number. Likewise, the subject's name will not appear in either reports or other publication of results from this study. In addition to participant information about participation in the trial, the test subjects will separately receive both verbal and written information about the processing and storage of their data.

Sharing of personal information/biological material abroad

Personal information will be shared through a centralized database, Sharepoint, hosted by the University of Limerick, which will be used for sharing the collected data and monitoring the different phases of the project with collaborating partners at Tel-Aviv University (Israel), University of Limerick (Ireland) and University of Cagliari (Italy). The Sharepoint database will be stored in the servers of the University of Limerick, which are protected by specific firewall and security protocols A unique ID will be created for each participant, which will ensure pseudonymisation, only the originating site will hold the key to the pseudonymisation. Data will be acquired in the software during clinical assessment by personnel logged with secure credentials in the Sharepoint system. Every involved center will have visibility to their own data only, with the exception of the coordinator that will be able to visualize pseudonymisation data from all centers. The data shared with the parties, is shared in order to be able to conduct analysis at the end of the project. We comply Danish Data Protection Act and with the GDPR rules both in Denmark and when data is transferred to third country. In accordance with the data transfer we comply with chapter 5. Noteworthy, Israel is considered a country outside of the EU with an adequate level of data protection. A Joint Controller Data Sharing Agreement has been made between the parties.

Economy

The initiative for the project has been taken by senior researcher Lars G. Hvid, together with international colleagues Massimiliano Pau (Italy), Susan Coote (Ireland) and Alon Kalron (Israel).

The project is funded by the Transforming Health and Care Systems (THCS), with 2.190.857 DKK granted to the Danish MS Hospitals and paid out to them. The funding is supporting salary for a researcher along with running costs of the project. Of note, all persons involved in the project work independently of the funders.

Recruitment of participants and informed consent

The MS clinic refers patients with a need for rehabilitation to the hospital as usual through the referral process, and the hospital's research department (i.e. the project leader) receives notification of potential participants. The initial contact is made by the project leader through an in-person interview, providing verbal information about the project. If the patient shows interest the written participant information "Deltagerinformation" will be sent together with the pamphlets "Før du beslutter dig - Om at være forsøgsperson i sundhedsvidenskabelige forsøg" and "Forsøgspersoners rettigheder i et sundhedsvidenskabeligt forskningsprojekt" (relates to the rights of participating in health research studies).

If the patient wishes to participate, a minimum of one hour is allocated for an oral information meeting with the project leader in an undisturbed room that has been booked in advance on the same day as the patient is admitted. The patient will be informed that they have the right to bring another person present at this physical information meeting at the hospital. At this meeting, the patients will be screened according to the inclusion and exclusion criteria. After the oral information meeting, the patient is offered time to sign the informed consent form, together with the project leader, and the participant will be offered a copy of the consent form, with a minimum reflection period of 24 hours. By signing, the participant gives his/hers consent to participate in the project.

The regular rehabilitation at the MS-hospital is conducted regardless of whether the patient chooses to participate in the project or not.

Circumstances for termination of project and for participants being excluded from the project

All participants involved in the project are free to withdraw their consent to participate at any time without giving further explanation. This will have no influence on their general treatment.

If serious side effects or unexpected risks to participants are discovered, the study may be terminated to protect their health and safety. We cannot imagine any further cases where circumstances could lead to termination of the project, but should this happen contrary to expectations, the subjects will be informed of the interruption and the reason for this.

If a participant (against all expectations) incurs a more serious injury or experience any discomfort caused by the tests or training, participation in the project will be discontinued. If unforeseen risks (minor inconveniences) arise in connection with the project, or if a participant do not wish to complete a test or training session, we will have a discussion with the participant about continued participation in the project.

If the inclusion criteria are not met, the subjects will not be included in the project. If adverse events occur, it will be discussed whether the participant should be excluded from the project (the participant has the last say in this).

Publication of results

The results from the project will be compiled into one or more articles aimed at peer-reviewed scientific journals. We expect that 2-3 publications will come out of projects. The authorship statement will follow the Vancouver declaration. Both positive, negative, and inconclusive results will be published. If participants want information about the project's results, this can be obtained by contacting the project leaders.

Scientific ethical considerations

As previously described, the limited risks associated with this research project do not give rise to any special ethical concerns, especially when weighed against the substantial benefits that may be associated with this project. In this project, participants will get the opportunity to improve their overall health on multiple parameters.

If the project is successful, it can help improve clinical practice and contribute significantly to the treatment of MS. This is also of socio-economic importance, as the project may contribute to a new MS treatment approach. Collectively, with the minimalistic risks associated with the included methods, the execution of the project is considered ethically responsible.

Information regarding indemnity scheme

This study is covered by the Danish Patient Compensation. In case any adverse events occur, this will be registered to the Danish Patient Compensation, and they will help decide on the matter.

Contact information

Project leader

Laurits Taul-Madsen
The Danish MS Hospitals, Ry and Haslev
Klostervej 136, 8680 Ry
lauman@sclerosehospital.dk
Phone +45 61656063

Project supervisor

Lars G. Hvid
The Danish MS Hospitals, Ry and Haslev
Klostervej 136, 8680 Ry
larshv@sclerosehospital.dk
Phone +45 93508717

1. Walton C, King R, Rechtman L, Kaye W, Leray E, Marrie RA, et al. Rising prevalence of multiple sclerosis worldwide: Insights from the Atlas of MS, third edition. Mult Scler. 2020;26(14):1816-21.
2. Heesen C, Böhm J, Reich C, Kasper J, Goebel M, Gold SM. Patient perception of bodily functions in multiple sclerosis: gait and visual function are the most valuable. Mult Scler. 2008;14(7):988-91.
3. van der Feen FE, de Haan GA, van der Lijn I, Heersema DJ, Meilof JF, Heutink J. Independent outdoor mobility of persons with multiple sclerosis - A systematic review. Mult Scler Relat Disord. 2020;37:101463.
4. Kalron A, Dvir Z, Achiron A. Walking while talking--difficulties incurred during the initial stages of multiple sclerosis disease process. Gait Posture. 2010;32(3):332-5.
5. Souza A, Kelleher A, Cooper R, Cooper RA, Iezzoni LI, Collins DM. Multiple sclerosis and mobility-related assistive technology: systematic review of literature. J Rehabil Res Dev. 2010;47(3):213-23.
6. Ezeugwu V, Klaren RE, E AH, Manns PT, Motl RW. Mobility disability and the pattern of accelerometer-derived sedentary and physical activity behaviors in people with multiple sclerosis. Prev Med Rep. 2015;2:241-6.
7. Sandroff BM, Klaren RE, Motl RW. Relationships among physical inactivity, deconditioning, and walking impairment in persons with multiple sclerosis. J Neurol Phys Ther. 2015;39(2):103-10.
8. Motl RW. Physical activity and irreversible disability in multiple sclerosis. Exerc Sport Sci Rev. 2010;38(4):186-91.
9. Schmidt S, Jöstingmeyer P. Depression, fatigue and disability are independently associated with quality of life in patients with multiple Sclerosis: Results of a cross-sectional study. Mult Scler Relat Disord. 2019;35:262-9.
10. Naci H, Fleurence R, Birt J, Duhig A. The impact of increasing neurological disability of multiple sclerosis on health utilities: a systematic review of the literature. J Med Econ. 2010;13(1):78-89.
11. Koch-Henriksen N, Magyari M. Apparent changes in the epidemiology and severity of multiple sclerosis. Nat Rev Neurol. 2021;17(11):676-88.
12. Dalgas U, Langeskov-Christensen M, Stenager E, Riemenschneider M, Hvid LG. Exercise as Medicine in Multiple Sclerosis-Time for a Paradigm Shift: Preventive, Symptomatic, and Disease-Modifying Aspects and Perspectives. Curr Neurol Neurosci Rep. 2019;19(11):88.

13. Motl RW, Sandroff BM. Benefits of Exercise Training in Multiple Sclerosis. Curr Neurol Neurosci Rep. 2015;15(9):62.
14. Latimer-Cheung AE, Pilutti LA, Hicks AL, Martin Ginis KA, Fenuta AM, MacKibbon KA, et al. Effects of exercise training on fitness, mobility, fatigue, and health-related quality of life among adults with multiple sclerosis: a systematic review to inform guideline development. Arch Phys Med Rehabil. 2013;94(9):1800-28.e3.
15. Casey B, Coote S, Shirazipour C, Hannigan A, Motl R, Martin Ginis K, et al. Modifiable Psychosocial Constructs Associated With Physical Activity Participation in People With Multiple Sclerosis: A Systematic Review and Meta-Analysis. Arch Phys Med Rehabil. 2017;98(7):1453-75.
16. Moumdjian L, Smedal T, Arntzen EC, van der Linden ML, Learmonth Y, Pedullà L, et al. Impact of the COVID-19 Pandemic on Physical Activity and Associated Technology Use in Persons With Multiple Sclerosis: An International RIMS-SIG Mobility Survey Study. Arch Phys Med Rehabil. 2022;103(10):2009-15.
17. Pedullà L, Santoyo-Medina C, Novotna K, Moumdjian L, Smedal T, Arntzen EC, et al. Physical Activity in Multiple Sclerosis: Meeting the Guidelines at the Time of the COVID-19 Pandemic. J Neurol Phys Ther. 2023;47(2):112-21.
18. Jonsdottir J, Santoyo-Medina C, Kahraman T, Kalron A, Rasova K, Moumdjian L, et al. Changes in physiotherapy services and use of technology for people with multiple sclerosis during the COVID-19 pandemic. Mult Scler Relat Disord. 2023;71:104520.
19. Truijen S, Abdullahi A, Bijsterbosch D, van Zoest E, Conijn M, Wang Y, et al. Effect of home-based virtual reality training and telerehabilitation on balance in individuals with Parkinson disease, multiple sclerosis, and stroke: a systematic review and meta-analysis. Neurol Sci. 2022;43(5):2995-3006.
20. Ghahfarrokhi MM, Banitalebi E, Negaresh R, Motl RW. Home-Based Exercise Training in Multiple Sclerosis: A Systematic Review with Implications for Future Research. Mult Scler Relat Disord. 2021;55:103177.
21. Thompson AJ, Banwell BL, Barkhof F, Carroll WM, Coetzee T, Comi G, et al. Diagnosis of multiple sclerosis: 2017 revisions of the McDonald criteria. Lancet Neurol. 2018;17(2):162-73.
22. Teresi JA, Yu X, Stewart AL, Hays RD. Guidelines for Designing and Evaluating Feasibility Pilot Studies. Med Care. 2022;60(1):95-103.
23. Parmanto B, Lewis AN, Jr., Graham KM, Bertolet MH. Development of the Telehealth Usability Questionnaire (TUQ). Int J Telerehabil. 2016;8(1):3-10.
24. McGuigan C, Hutchinson M. Confirming the validity and responsiveness of the Multiple Sclerosis Walking Scale-12 (MSWS-12). Neurology. 2004;62(11):2103-5.
25. Feng Y-S, Kohlmann T, Janssen MF, Buchholz I. Psychometric properties of the EQ-5D-5L: a systematic review of the literature. Quality of Life Research. 2021;30(3):647-73.

26. Riazi A, Hobart JC, Lamping DL, Fitzpatrick R, Thompson AJ. Multiple Sclerosis Impact Scale (MSIS-29): reliability and validity in hospital based samples. J Neurol Neurosurg Psychiatry. 2002;73(6):701-4.
27. Larson RD. Psychometric properties of the modified fatigue impact scale. Int J MS Care. 2013;15(1):15-20.
28. Julian LJ. Measures of anxiety: State-Trait Anxiety Inventory (STAI), Beck Anxiety Inventory (BAI), and Hospital Anxiety and Depression Scale-Anxiety (HADS-A). Arthritis Care & Research. 2011;63(S11):S467-S72.
29. Rush AJ, Bernstein IH, Trivedi MH, Carmody TJ, Wisniewski S, Mundt JC, et al. An evaluation of the quick inventory of depressive symptomatology and the hamilton rating scale for depression: a sequenced treatment alternatives to relieve depression trial report. Biol Psychiatry. 2006;59(6):493-501.
30. Beecham J, Knapp M. Costing psychiatric interventions. Measuring mental health needs: Gaskell/Royal College of Psychiatrists; 1992. p. 163-83.
31. Zheng P, Huynh TLT, Jones CD, Feasel CD, Jeng B, Motl RW. Validity of the 30-Second Sit-to-Stand test as a measure of lower extremity function in persons with multiple sclerosis: Preliminary evidence. Multiple Sclerosis and Related Disorders. 2023;71.
32. Franchignoni F, Horak F, Godi M, Nardone A, Giordano A. Using psychometric techniques to improve the Balance Evaluation Systems Test: the mini-BESTest. J Rehabil Med. 2010;42(4):323-31.
33. Soke F, Eldemir S, Ozkan T, Ozkul C, Ozcan Gulsen E, Gulsen C, et al. The functional reach test in people with multiple sclerosis: a reliability and validity study. Physiother Theory Pract. 2022;38(13):2905-19.
34. Motl RW, Learmonth YC, Wójcicki TR, Fanning J, Hubbard EA, Kinnett-Hopkins D, et al. Preliminary validation of the short physical performance battery in older adults with multiple sclerosis: secondary data analysis. BMC Geriatr. 2015;15:157.
35. Kieseier BC, Pozzilli C. Assessing walking disability in multiple sclerosis. Mult Scler. 2012;18(7):914-24.
36. Benedict RH, DeLuca J, Phillips G, LaRocca N, Hudson LD, Rudick R. Validity of the Symbol Digit Modalities Test as a cognition performance outcome measure for multiple sclerosis. Mult Scler. 2017;23(5):721-33.
