# Supplementary material for: Protocol for a pilot effectiveness and feasibility randomized controlled trial of an advanced telerehabilitation system for people with multiple sclerosis (PLATINUMS project)
Source: PLoS One. 2026 Aug 18;21(8):e0355376. doi: 10.1371/journal.pone.0355376 (PMC13485066; doi:10.1371/journal.pone.0355376)
Supplement: Supplementary file 2 [file pone.0355376.s002.docx]

**Figure 1: Participant timeline- schedule of enrollment, interventions, and assessments.**

|  | **Screening** | **Baseline assessment** | ***10 week  intervention period*** | ***Follow-up***  ***assessment*** |
| --- | --- | --- | --- | --- |
| **ENROLLMENT:** |  |  |  |  |
| **Eligibility screen** | X |  |  |  |
| **Informed consent** | X |  |  |  |
| **Randomization** |  | X |  |  |
| **INTERVENTION/ COMPARATOR:** |  |  |  |  |
| ***[AI Telerehabilitation]*** |  |  | X |  |
| ***[Paper-based exercises]*** |  |  | X |  |
| **ASSESSMENTS:** |  |  |  |  |
|  | **Mobility** | | | |
| ***30 sec sit to stand*** |  | X |  | X |
| ***SPPB*** |  | X |  | X |
| ***FRT*** |  | X |  | X |
| ***T25FWT*** |  | X |  | X |
| ***MINIBESTest*** |  | X |  | X |
| ***6MWT*** |  | X |  | X |
|  | **Cognition** | | | |
| ***SDMT*** |  | X |  |  |
|  | **PROs** | | | |
| ***MSIS-29*** |  | X |  | X |
| ***MFIS*** |  | X |  | X |
| ***MSWS-12*** |  | X |  | X |
| ***EQ-5D-5L*** |  | X |  | X |
| ***STAI*** |  | X |  | X |
| ***QIDS*** |  | X |  | X |
| ***CSRI*** |  | X |  | X |
| ***TUQ*** |  |  |  | X |
| ***SSI*** |  |  |  | X |

Abbreviations: SPPB: Short Physical Performance Battery, FRT: Functional Reach Test, T25FWT: Timed 25 Feet Foot Walk Test, MINIBEStest: Mini Balance Evaluation Systems Test, 6MWT: Six-minute Walk Test, SDMT: Symbol Digit Modalities Test, MSIS-29: 29-Item Multiple Sclerosis Impact Scale, MFIS: Modified Fatigue Impact Scale, MSWS-12: 12-Item Multiple Sclerosis Walking Scale, EQ-5D-5L: EuroQol 5 Dimensions 5 Levels, STAI: State-Trait Anxiety Inventory, QIDS: Quick Inventory of Depressive Symptomatology, TUQ: Telehealth Usability Questionnaire, SSI: Semi-structured interview.
