# Supplementary material for: Protocol for a pilot effectiveness and feasibility randomized controlled trial of an advanced telerehabilitation system for people with multiple sclerosis (PLATINUMS project)
Source: PLoS One. 2026 Aug 18;21(8):e0355376. doi: 10.1371/journal.pone.0355376 (PMC13485066; doi:10.1371/journal.pone.0355376)
Supplement: Peer review history

# Peer review: Decision Letter 0

**DOI:** 10.1371/journal.pone.0355376.r001

**Authors:** Imre Cikajlo

29 May 2026

Dear Dr. Madsen,

Thank you for submitting your manuscript to PLOS ONE. After careful consideration, we feel that it has merit but does not fully meet PLOS ONE’s publication criteria as it currently stands. Therefore, we invite you to submit a revised version of the manuscript that addresses the points raised during the review process.

Please submit your revised manuscript by Jul 13 2026 11:59PM. If you will need more time than this to complete your revisions, please reply to this message or contact the journal office at plosone@plos.org. When you're ready to submit your revision, log on to https://www.editorialmanager.com/pone/ and select the 'Submissions Needing Revision' folder to locate your manuscript file.

- A letter that responds to each point raised by the academic editor and reviewer(s). You should upload this letter as a separate file labeled 'Response to Reviewers'.
- A marked-up copy of your manuscript that highlights changes made to the original version. You should upload this as a separate file labeled 'Revised Manuscript with Track Changes'.
- An unmarked version of your revised paper without tracked changes. You should upload this as a separate file labeled 'Manuscript'.

If you would like to make changes to your financial disclosure, please include your updated statement in your cover letter. Guidelines for resubmitting your figure files are available below the reviewer comments at the end of this letter.

If applicable, we recommend that you deposit your laboratory protocols in protocols.io to enhance the reproducibility of your results. Protocols.io assigns your protocol its own identifier (DOI) so that it can be cited independently in the future. For instructions see: https://journals.plos.org/plosone/s/submission-guidelines#loc-laboratory-protocols. Additionally, PLOS ONE offers an option for publishing peer-reviewed Lab Protocol articles, which describe protocols hosted on protocols.io. Read more information on sharing protocols at https://plos.org/protocols?utm_medium=editorial-email&utm_source=authorletters&utm_campaign=protocols.

As the corresponding author, your ORCID iD is verified in the submission system and will appear in the published article. PLOS supports the use of ORCID, and we encourage all coauthors to register for an ORCID iD and use it as well. Please encourage your coauthors to verify their ORCID iD within the submission system before final acceptance, as unverified ORCID iDs will not appear in the published article. *Only*  the individual author can complete the verification step; PLOS staff *cannot*  verify ORCID iDs on behalf of authors.

We look forward to receiving your revised manuscript.

Kind regards,

Imre Cikajlo, Ph.D.

Academic Editor

PLOS One

**Journal Requirements:**

When submitting your revision, we need you to address these additional requirements.

1. Please ensure that your manuscript meets PLOS ONE's style requirements, including those for file naming. The PLOS ONE style templates can be found at

https://journals.plos.org/plosone/s/file?id=wjVg/PLOSOne_formatting_sample_main_body.pdf and

https://journals.plos.org/plosone/s/file?id=ba62/PLOSOne_formatting_sample_title_authors_affiliations.pdf

2. Thank you for stating the following financial disclosure:

“The study is funded by the European Partnership on Transforming Health and Care Systems (THCS) through the Horizon Europe Framework Program for Research and Innovation (2021-2027) and the joint Translational call 2023, with grant number 1538.”

Please state what role the funders took in the study.  If the funders had no role, please state: "The funders had no role in study design, data collection and analysis, decision to publish, or preparation of the manuscript."

If this statement is not correct you must amend it as needed.

Please include this amended Role of Funder statement in your cover letter; we will change the online submission form on your behalf.

3. Please note that funding information should not appear in any section or other areas of your manuscript. We will only publish funding information present in the Funding Statement section of the online submission form. Please remove any funding-related text from the manuscript.

4. Please include captions for your Supporting Information files at the end of your manuscript, and update any in-text citations to match accordingly. Please see our Supporting Information guidelines for more information: http://journals.plos.org/plosone/s/supporting-information.

5. If the reviewer comments include a recommendation to cite specific previously published works, please review and evaluate these publications to determine whether they are relevant and should be cited. There is no requirement to cite these works unless the editor has indicated otherwise.

[Note: HTML markup is below. Please do not edit.]

Reviewers' comments:

Reviewer's Responses to Questions

**Comments to the Author**

1. Does the manuscript provide a valid rationale for the proposed study, with clearly identified and justified research questions?

Reviewer #1: Yes

Reviewer #2: Yes

**********

2. Is the protocol technically sound and planned in a manner that will lead to a meaningful outcome and allow testing the stated hypotheses?

Reviewer #1: Partly

Reviewer #2: Yes

**********

3. Is the methodology feasible and described in sufficient detail to allow the work to be replicable?

Reviewer #1: Yes

Reviewer #2: Yes

**********

4. Have the authors described where all data underlying the findings will be made available when the study is complete?

The PLOS Data policy

Reviewer #1: Yes

Reviewer #2: No

**********

5. Is the manuscript presented in an intelligible fashion and written in standard English?

Reviewer #1: Yes

Reviewer #2: Yes

**********

Please use the space provided to explain your answers to the questions above and, if applicable, provide comments about issues authors must address before this protocol can be accepted for publication. You may also include additional comments for the author, including concerns about research or publication ethics.

You may also provide optional suggestions and comments to authors that they might find helpful in planning their study.

Reviewer #1: This study is a multinational (4 institutions), feasibility, pilot randomized controlled trial investigating the effects of an AI-based telerehabilitation intervention in persons with multiple sclerosis (pwMS). Participants (n=96) will be randomized to receive either 10 weeks of a home-based AI telerehabilitation intervention or a home-based exercise intervention with paper-based instructions. The primary clinical outcome is the 30-second sit-to-stand test (30STS), and secondary clinical outcomes are balance, fatigue, MS impact, quality of life, symptoms of anxiety and depression and cost effectiveness analyses. Feasibility, in terms of recruitment and retention, acceptability of procedures and compliance and adherence will also be captured. The document has been well planned and written clearly.

There are some further considerations needed.

1. One needs a reasonable retention strategy to be spelled out more specifically for this special population.

2. One needs a data quality control and data monitoring strategy among the 4 institutions. They note that regular data checks will be performed to ensure completeness and accuracy of data. Please be specific.

3. Since this is a feasibility study there is no formal sample size power considerations. However, 96 subjects randomized between the two arm appears sufficient according to the guidelines they have referenced. Again pay attention to retention.

The primary analysis strategy to examine changes in outcomes from baseline to the end of the intervention period, a multivariate repeated-measures mixed-effects regression model will be applied. Participant ID will be included as a random effect, and group (intervention vs. comparator) and time (T0 vs. T1) will be included as fixed effects. The ITT approach will be verified by the Per protocol analysis. Please be sure these (ITT, PP) are defined appropriately for this kind of study.

In the Statistical analysis section the authors note that the model's assumptions, including normality, homogeneity of variance, and normally distributed residuals, will be assessed. If these assumptions are not met, appropriate data transformations will be applied.

4. They should also consider any multivariate non parametric options for non-normal data analysis.

5. Economic data capture forms should be in the supplement or appendix and reviewed by an economic expert for content and procedure.

6. Sensitivity analyses will be performed to evaluate the robustness of the findings under various assumptions regarding missing data. Please be specific. Also how are you defining robustness in this context?

7. The statistical feasibility analysis strategy should be clearly presented.

8. The strengths and limitations appear to have been thought out and seem reasonable.

Reviewer #2: Overall, this is a clearly organized multicentre pilot RCT protocol with many core procedures in place. There are several key operational details missing or inconsistent, limiting reproducibility and risk-of-bias appraisal. In addition, the manuscript provides only a high-level description of the AI platform (camera-based motion analysis and real-time feedback) without adequate procedural/background detail on what the AI measures/outputs, how feedback is generated, and what validation evidence supports these measures; expanding this section and briefly situating the approach within the emerging literature on AI-enabled telerehabilitation/AI-guided exercise programs would strengthen transparency and interpretability.

1. The manuscript states de-identified data will be available “upon reasonable request,” which is typically insufficient for PLOS ONE protocols. Please provide a concrete, procedure-level plan for how and where de-identified data will be shared upon study completion (e.g., named repository, timing, access conditions).

2. "Sealed envelope procedure” is not enough for reproducibility. Please specify (a) how the random sequence is generated (computer-generated? block sizes? varying blocks?), (b) whether randomization is stratified, and (c) how concealment is protected (who prepares/holds envelopes, tamper-proofing, when opened).

3. The protocol notes the T0 evaluator is blinded, but it is unclear who performs T1 assessments and how blinding will be maintained. Please explicitly describe assessor blinding at T1 and procedures to minimize unblinding (separate staff, standardized scripts, participant instructions).

4. Exercise selection is described as guideline-based plus therapist clinical reasoning, but the protocol lacks operational standardization/fidelity procedures. Please add minimum required exercise domains, progression rules, therapist training/certification, and fidelity checks (e.g., audits of prescriptions/logs across sites).

**********

what does this mean?). If published, this will include your full peer review and any attached files.

If you choose “no”, your identity will remain anonymous but your review may still be made public.

**Do you want your identity to be public for this peer review?**  For information about this choice, including consent withdrawal, please see our Privacy Policy

Reviewer #1: No

Reviewer #2: **Yes:** Jereme D. Wilroy

**********

[NOTE: If reviewer comments were submitted as an attachment file, they will be attached to this email and accessible via the submission site. Please log into your account, locate the manuscript record, and check for the action link "View Attachments". If this link does not appear, there are no attachment files.]

To ensure your figures meet our technical requirements, please review our figure guidelines: https://journals.plos.org/plosone/s/figures

You may also use PLOS’s free figure tool, NAAS, to help you prepare publication quality figures: https://journals.plos.org/plosone/s/figures#loc-tools-for-figure-preparation.

NAAS will assess whether your figures meet our technical requirements by comparing each figure against our figure specifications.

# Author comment: Author response to Decision Letter 1

**DOI:** 10.1371/journal.pone.0355376.r002

24 Jun 2026

Journal Requirements

1. Please ensure that your manuscript meets PLOS ONE's style requirements, including those for file naming.

We have reviewed the manuscript and supporting files and ensured that they comply with PLOS ONE formatting and file-naming requirements.

2. Thank you for stating the following financial disclosure:

“The study is funded by the European Partnership on Transforming Health and Care Systems (THCS) through the Horizon Europe Framework Program for Research and Innovation (2021-2027) and the joint Translational call 2023, with grant number 1538.”

Please state what role the funders took in the study. If the funders had no role, please state: "The funders had no role in study design, data collection and analysis, decision to publish, or preparation of the manuscript."

If this statement is not correct you must amend it as needed.

Please include this amended Role of Funder statement in your cover letter; we will change the online submission form on your behalf.

The statement has been added to the main manuscript

Lines 28-30

3. Please note that funding information should not appear in any section or other areas of your manuscript. We will only publish funding information present in the Funding Statement section of the online submission form. Please remove any funding-related text from the manuscript.

The manuscript does not include any funding-related text, excluding the Funding statement section.

4. Please include captions for your Supporting Information files at the end of your manuscript, and update any in-text citations to match accordingly.

Captions for supporting information has now been added to the manuscript.

5. If the reviewer comments include a recommendation to cite specific previously published works, please review and evaluate these publications to determine whether they are relevant and should be cited. There is no requirement to cite these works unless the editor has indicated otherwise.

Both reviewers' comments did not include a recommendation to cite specific published works

Reviewer #1:

This study is a multinational (4 institutions), feasibility, pilot randomized controlled trial investigating the effects of an AI-based telerehabilitation intervention in persons with multiple sclerosis (pwMS). Participants (n=96) will be randomized to receive either 10 weeks of a home-based AI telerehabilitation intervention or a home-based exercise intervention with paper-based instructions. The primary clinical outcome is the 30-second sit-to-stand test (30STS), and secondary clinical outcomes are balance, fatigue, MS impact, quality of life, symptoms of anxiety and depression and cost effectiveness analyses. Feasibility, in terms of recruitment and retention, acceptability of procedures and compliance and adherence will also be captured. The document has been well planned and written clearly.

We thank the reviewer for his/her positive feedback

1. One needs a reasonable retention strategy to be spelled out more specifically for this special population.

Thank you for this comment.

We agree that retention is an important consideration in people with MS. We have added a description of the retention strategies used in this study, including regular participant contact, flexible scheduling, technical support, and individualized follow-up throughout the intervention period.

Now reads: (Lines 194-198)

"To promote retention, participants will receive regular contact throughout the study, including weekly text messages and scheduled telephone calls. Flexible scheduling of assessments, individualized exercise progression, and technical support for the telerehabilitation platform will be provided as needed."

2. One needs a data quality control and data monitoring strategy among the 4 institutions. They note that regular data checks will be performed to ensure completeness and accuracy of data. Please be specific.

Thank you for this comment.

We have revised the manuscript to provide a more detailed description of the data quality control and monitoring procedures, including centralized oversight, regular data reviews, use of the consortium SharePoint platform, and processes to ensure data completeness and consistency across all study sites.

Now reads: (Lines 348-355)

"…Data quality monitoring will be coordinated centrally by S.C., who leads the project's data management work package. Regular consortium meetings and Trial Management Committee/Advisory Board meetings will be held to review data quality and protocol adherence across all participating centers. All procedures will be conducted in accordance with the project Data Management Plan and Joint Controller Agreement. Data for each country will be held locally using relevant procedures and pseudonymized data will be entered into the consortium SharePoint platform held by UL, which serves as the secure central repository for the study. Monthly reviews will be conducted to identify missing data, inconsistencies, out-of-range values, and protocol deviations. Any queries will be communicated to the relevant site for clarification or correction against source data."

3. Since this is a feasibility study there is no formal sample size power considerations. However, 96 subjects randomized between the two arm appears sufficient according to the guidelines they have referenced. Again pay attention to retention. The primary analysis strategy to examine changes in outcomes from baseline to the end of the intervention period, a multivariate repeated-measures mixed-effects regression model will be applied. Participant ID will be included as a random effect, and group (intervention vs. comparator) and time (T0 vs. T1) will be included as fixed effects. The ITT approach will be verified by the Per protocol analysis. Please be sure these (ITT, PP) are defined appropriately for this kind of study.

In the Statistical analysis section the authors note that the model's assumptions, including normality, homogeneity of variance, and normally distributed residuals, will be assessed. If these assumptions are not met, appropriate data transformations will be applied.

Thank you for this comment.

We have revised the Statistical Analysis Plan to further clarify the definitions of the intention-to-treat and per-protocol analyses. We have also provided additional detail regarding the assessment of model assumptions and the planned approach should these assumptions not be met.

Now reads: (Lines 368-371; 374-376)

"… The model's assumptions, including normality of residuals and homogeneity of variance, will be assessed using graphical and statistical methods. If these assumptions are not met, appropriate data transformations, non-parametric methods, or other alternative statistical approaches will be considered.

…A secondary per-protocol analysis will be conducted, including participants who complete the post-intervention assessment and meet the predefined adherence (≥80 %) and compliance (≥75%) criteria."

4. They should also consider any multivariate non-parametric options for non-normal data analysis.

Thank you for this suggestion.

We agree that alternative analytical approaches may be required if model assumptions are not met. We have revised the Statistical Analysis section to indicate that, in addition to data transformation, appropriate nonparametric methods will be considered when warranted by the data distribution.

5. Economic data capture forms should be in the supplement or appendix and reviewed by an economic expert for content and procedure.

Thank you for this comment.

The economic data capture forms have been added to the supplementary material. Furthermore, we have adapted the section so it now reads. Lines 271-274. “The economic evaluation methodology, data collection procedures, and resource-use measures were developed with input and guidance from a health economics expert within the PLATINUMS consortium to ensure the appropriateness of the proposed approach.”

6. Sensitivity analyses will be performed to evaluate the robustness of the findings under various assumptions regarding missing data. Please be specific. Also how are you defining robustness in this context?

Thank you for this comment.

We have revised the Statistical Analysis section to provide additional detail regarding the planned sensitivity analyses. Specifically, sensitivity analyses will compare the primary findings obtained from the intention-to-treat and per-protocol populations and will assess the potential impact of missing outcome data. In this context, robustness refers to the consistency of the overall conclusions across different analytical assumptions.

Now reads: (Lines 374-377)

"Sensitivity analyses will be performed to assess the impact of missing outcome data and to compare findings obtained from the intention-to-treat and per-protocol populations. Robustness will be evaluated by examining whether the overall direction and interpretation of the results remain consistent across these analyses."

7. The statistical feasibility analysis strategy should be clearly presented.

Thank you for this comment.

We agree that the analysis of feasibility outcomes should be more explicitly described. We have revised the Statistical Analysis section to clarify how recruitment, retention, adherence, compliance, safety, and acceptability outcomes will be summarized and evaluated.

Now reads: (Lines 357-361)

"Feasibility outcomes, including recruitment rate, retention rate, adherence, compliance, acceptability, and adverse events, will be summarized using descriptive statistics. Continuous variables will be reported as means and standard deviations or medians and interquartile ranges, as appropriate, while categorical variables will be reported as frequencies and percentages. These outcomes will be used to assess the feasibility of conducting a future definitive trial."

8. The strengths and limitations appear to have been thought out and seem reasonable.

We thank the reviewer for this positive feedback.

Reviewer #2:

1. Overall, this is a clearly organized multicentre pilot RCT protocol with many core procedures in place. There are several key operational details missing or inconsistent, limiting reproducibility and risk-of-bias appraisal. In addition, the manuscript provides only a high-level description of the AI platform (camera-based motion analysis and real-time feedback) without adequate procedural/background detail on what the AI measures/outputs, how feedback is generated, and what validation evidence supports these measures; expanding this section and briefly situating the approach within the emerging literature on AI-enabled telerehabilitation/AI-guided exercise programs would strengthen transparency and interpretability.

We thank the reviewer for the positive assessment of the protocol and for these helpful suggestions.

We have expanded the description of the AI-enabled telerehabilitation platform to provide additional detail regarding its movement-tracking capabilities, real-time feedback mechanisms, and clinical application. We have also added contextual information regarding the emerging role of AI-supported telerehabilitation in rehabilitation practice to improve transparency and interpretability.

Now reads: (Lines 221-228)

"Using computer vision technology, MoveAI tracks participant movements during exercise and compares performance against predefined movement parameters. The system provides real-time feedback regarding exercise execution, movement quality, range of motion, and repetition completion, thereby optimising movement performance. In addition, exercise completion and engagement metrics are automatically recorded, allowing clinicians to remotely monitor adherence and progression. AI-enabled telerehabilitation systems have emerged as a promising approach to enhance accessibility, personalization, and monitoring of rehabilitation interventions delivered in the home environment."

1. The manuscript states de-identified data will be available “upon reasonable request,” which is typically insufficient for PLOS ONE protocols. Please provide a concrete, procedure-level plan for how and where de-identified data will be shared upon study completion (e.g., named repository, timing, access conditions).

We have revised the Data Availability Statement to provide a more specific data-sharing plan, including public availability of the de-identified dataset and supporting documentation following publication of the primary study results.

Now reads: (Lines 34-37)

"De-identified participant-level data and supporting documentation will be made publicly available in an appropriate data repository following publication of the primary study results, subject to applicable ethical, legal, and data protection requirements. Details of the repository and access information will be provided upon completion of the study."

2. "Sealed envelope procedure” is not enough for reproducibility. Please specify (a) how the random sequence is generated (computer-generated? block sizes? varying blocks?), (b) whether randomization is stratified, and (c) how concealment is protected (who prepares/holds envelopes, tamper-proofing, when opened).

Thank you for this comment.

We have revised the manuscript to provide additional detail on the randomization procedure, including the generation of the allocation sequence, stratification by study site, and the procedures used to ensure allocation concealment.

Now reads: (Lines 169-174)

"A computer-generated randomization sequence stratified by study site will be used. Randomization procedures at all sites will be coordinated according to a common study protocol developed by the project team. Allocation concealment will be ensured using sequentially numbered, opaque, sealed envelopes prepared and maintained locally at each participating site. Envelopes will be opened only after eligibility has been confirmed and baseline assessments have been completed."

3. The protocol notes the T0 evaluator is blinded, but it is unclear who performs T1 assessments and how blinding will be maintained. Please explicitly describe assessor blinding at T1 and procedures to minimize unblinding (separate staff, standardized scripts, participant instructions).

Thank you for this comment.

We have revised the manuscript to clarify assessor blinding procedures at both baseline and follow-up assessments, including measures implemented to minimize the risk of unblinding throughout the study.

Now reads: (Lines 335-339)

"Outcome assessments at both T0 and T1 will be conducted by assessors blinded to group allocation whenever possible. Participants will be instructed not to disclose their treatment allocation during assessments. Standardized assessment procedures will be used across sites. Any instances of unblinding will be documented."

4. Exercise selection is described as guideline-based plus therapist clinical reasoning, but the protocol lacks operational standardization/fidelity procedures. Please add minimum required exercise domains, progression rules, therapist training/certification, and fidelity checks (e.g., audits of prescriptions/logs across sites).

Thank you for this comment.

We agree that additional detail regarding intervention standardization is warranted. We have revised the manuscript to further describe the exercise prescription framework, progression procedures, therapist training, and measures implemented to promote intervention fidelity across participating sites.

Now reads: (Lines 208-215)

"To ensure consistency across sites, exercise prescription will follow a common intervention framework developed by the consortium. Exercise programs will include a combination of lower-limb strengthening, balance, and mobility exercises, with individual tailoring based on participants' functional status. Progression of exercises will be guided by participant performance, perceived exertion, and safety considerations. Prior to study initiation, all physiotherapists will receive training on the study procedures and intervention framework. Intervention fidelity will be supported through regular consortium meetings and periodic review of exercise prescriptions and participant logs across sites."

# Peer review: Decision Letter 1

**DOI:** 10.1371/journal.pone.0355376.r003

**Authors:** Imre Cikajlo

21 Jul 2026

Protocol for a pilot effectiveness and feasibility randomized controlled trial of an advanced telerehabilitation system for people with multiple sclerosis (PLATINUMS project).

PONE-D-26-10480R1

Dear Dr. Madsen,

We’re pleased to inform you that your manuscript has been judged scientifically suitable for publication and will be formally accepted for publication once it meets all outstanding technical requirements.

Within one week, you’ll receive an e-mail detailing the required amendments. When these have been addressed, you’ll receive a formal acceptance letter and your manuscript will be scheduled for publication.

An invoice will be generated when your article is formally accepted. Please note, if your institution has a publishing partnership with PLOS and your article meets the relevant criteria, all or part of your publication costs will be covered. Please make sure your user information is up-to-date by logging into Editorial Manager at Editorial Manager® and clicking the ‘Update My Information' link at the top of the page. For questions related to billing, please contact billing support.

If your institution or institutions have a press office, please notify them about your upcoming paper to help maximize its impact. If they’ll be preparing press materials, please inform our press team as soon as possible -- no later than 48 hours after receiving the formal acceptance. Your manuscript will remain under strict press embargo until 2 pm Eastern Time on the date of publication. For more information, please contact onepress@plos.org.

Kind regards,

Imre Cikajlo, Ph.D.

Academic Editor

PLOS One

Additional Editor Comments (optional):

Reviewers' comments:

Reviewer's Responses to Questions

**Comments to the Author**

1. Does the manuscript provide a valid rationale for the proposed study, with clearly identified and justified research questions?

Reviewer #1: Yes

Reviewer #2: Yes

**********

2. Is the protocol technically sound and planned in a manner that will lead to a meaningful outcome and allow testing the stated hypotheses?

Reviewer #1: Yes

Reviewer #2: Yes

**********

3. Is the methodology feasible and described in sufficient detail to allow the work to be replicable?

Reviewer #1: Yes

Reviewer #2: Yes

**********

4. Have the authors described where all data underlying the findings will be made available when the study is complete?

The PLOS Data policy

Reviewer #1: Yes

Reviewer #2: Yes

**********

5. Is the manuscript presented in an intelligible fashion and written in standard English?

Reviewer #1: Yes

Reviewer #2: Yes

**********

Please use the space provided to explain your answers to the questions above and, if applicable, provide comments about issues authors must address before this protocol can be accepted for publication. You may also include additional comments for the author, including concerns about research or publication ethics.

You may also provide optional suggestions and comments to authors that they might find helpful in planning their study.

Reviewer #1: All comments have been addressed and the revisions in the manuscript are noted.

XXXXXXXXXXXXXXXXXXXXXXXXXXXXXXXXXXXXXXXXXXXXXXXXXXXXXXXXXXXXXXXXXXXXXXXXXXXXXXXXXXXXXXXXXXXXXXXXXXXXXXXXXXXXXXXXXXXXXXXXXXX

Reviewer #2: I thank the authors for their thorough and thoughtful response to my comments. The revisions and clarifications provided adequately address the concerns I raised in my initial review, and I am satisfied with how the critiques have been handled. I have no further concerns at this time and support the paper moving forward.

**********

what does this mean?). If published, this will include your full peer review and any attached files.

If you choose “no”, your identity will remain anonymous but your review may still be made public.

**Do you want your identity to be public for this peer review?**  For information about this choice, including consent withdrawal, please see our Privacy Policy

Reviewer #1: No

Reviewer #2: **Yes:** Jereme Wilroy

**********

# Editor report: Acceptance letter

**DOI:** 10.1371/journal.pone.0355376.r004

**Authors:** Imre Cikajlo

PONE-D-26-10480R1

PLOS One

Dear Dr. Taul-Madsen,

I'm pleased to inform you that your manuscript has been deemed suitable for publication in PLOS One. Congratulations! Your manuscript is now being handed over to our production team.

At this stage, our production department will prepare your paper for publication. This includes ensuring the following:

* All references, tables, and figures are properly cited

* All relevant supporting information is included in the manuscript submission,

* There are no issues that prevent the paper from being properly typeset

You will receive further instructions from the production team, including instructions on how to review your proof when it is ready. Please keep in mind that we are working through a large volume of accepted articles, so please give us a few days to review your paper and let you know the next and final steps.

Lastly, if your institution or institutions have a press office, please let them know about your upcoming paper now to help maximize its impact. If they'll be preparing press materials, please inform our press team within the next 48 hours. Your manuscript will remain under strict press embargo until 2 pm Eastern Time on the date of publication. For more information, please contact onepress@plos.org.

You will receive an invoice from PLOS for your publication fee after your manuscript has reached the completed accept phase. If you receive an email requesting payment before acceptance or for any other service, this may be a phishing scheme. Learn how to identify phishing emails and protect your accounts at https://explore.plos.org/phishing.

If we can help with anything else, please email us at customercare@plos.org.

Thank you for submitting your work to PLOS ONE and supporting open access.

Kind regards,

PLOS ONE Editorial Office Staff

on behalf of

Professor Imre Cikajlo

Academic Editor

PLOS One
